# Identification of the Production of Small Holes and Threads Using Progressive Technologies in Austenite Stainless Steel 1.4301

**DOI:** 10.3390/ma16196538

**Published:** 2023-10-03

**Authors:** Dana Stančeková, Filip Turian, Michal Šajgalík, Mário Drbúl, Nataša Náprstková, Anna Rudawská, Miroslav Špiriak

**Affiliations:** 1 Faculty of Mechanical Engineering, University of Zilina, Univerzitná 8215/1, 01026 Žilina, Slovakia; filip_turian@elmaxzilina.sk (F.T.); michal.sajgalik@fstroj.uniza.sk (M.Š.); mario.drbul@fstroj.uniza.sk (M.D.); spirimro@schaeffler.com (M.Š.); 2 Faculty of Mechanical Engineering, University J. E. Purkyně in Ústí nad Labem, Pasteurova 3544/1, 400 96 Ústí nad Labem, Czech Republic; natasa.naprstkova@ujep.cz; 3 Faculty of Mechanical Engineering, Lublin University of Technology, 20-618 Lublin, Poland; a.rudawska@pollub.pl

**Keywords:** thread, hole, cutting, laser, quality, shape accuracy

## Abstract

This article focuses on the technologies used by a manufacturing company to produce threads in chrome–nickel steel 1.4301 at specific sheet thicknesses. To enhance production quality, two specific technologies were chosen for hole formation, considering the requirements of the company. Both conventional drilling and nonconventional laser cutting methods were evaluated as potential techniques for hole production. Conventional thread-cutting technology and progressive forming technology were employed to create metric internal threads. The aim of integrating these diverse technologies is to identify the optimal solution for a specific sheet thickness in order to prevent the occurrence of defective threads that could not fulfil the intended purpose. The evaluation of the threads and holes relies on the examination of surface characteristics, such as the quality of the surface, as well as the lack of any signs of damage, cracks, or burrs. Furthermore, residual stresses in the surface layer were monitored because these stresses have the potential to cause cracking. Additionally, extensive monitoring was performed to guarantee that the form and size of the manufactured threads were correct to ensure smooth assembly and optimal functionality.

## 1. Introduction

As numerous authors in the field of engineering have noted, designers and production engineers frequently face the challenge of producing highly durable connections capable of facilitating the assembly and disassembly of components made of various materials. To fulfil this task, fasteners and internal threads represent the prevailing choices, as references [1,2] indicate. Threaded connections are one of the most prevalent solutions for assembling mechanical components because they result in assemblies with high strength and stiffness, as substantiated by references [1,3]. While screw connections find applications in technical structures, it is worth emphasizing that a substantial proportion of mechanical, aerospace, medical, and dental products incorporate at least one threaded component, as documented in references [4,5,6]. This underlines the versatility and practicality of threaded connections, further enhanced by their notably rapid component assembly and disassembly, as denoted in reference [7].

Historically, the use of nuts was the most common approach for ensuring the stability of mechanical components. However, in other circumstances, the direct integration of internal threads onto the joint component is used as an alternative method of fastening. According to the data reported in reference [5], the evaluation of mechanical stresses experienced by machine components demonstrates that internal threads are continuously subjected to tensile forces. According to Liu [8], the continued use of threads is bound to cause gradual deterioration, which may grow in intensity and demonstrate a correlation with the overall quality of the thread connections. It is crucial to note that the failure of fastening mechanisms, both permanent and non-permanent, can have significant consequences for the entire operation of mechanical systems, as cited in reference [9].

In their work, Val A.G.D. and colleagues [10] emphasise that the process of thread cutting is a multifaceted operation focused on the creation of internal openings for screws. This complexity stems from the pivotal need for precise synchronization between the rotational motion of the tap and the vertical movement, as well as for substantial contact between the tool and the workpiece. This synchronization and contact are especially critical when threading is performed under demanding cutting conditions. The production of internal threads typically involves one of three methods: cutting, cold forming, or milling. Thread cutting and thread milling represent conventional metal cutting techniques, whereas the cold forming of threads provides an alternative method that eliminates chip generation [11].

During the process of thread cutting, the cutting edges of the tap progressively shape the internal thread, which is a continual process. An integrated thread is produced by a single, complete thread-cutting process [12]. The requirements of thread cutting are high, primarily due to the critical need for precise synchronization between the rotational and vertical movements [1].

Threads formed through the thread-forming process are the result of plastic deformation and material displacement. In their study, Masmoudi, N. et al. [13] expound on the numerous advantages offered by the cold-forming thread process in comparison to conventional cutting techniques. However, it is essential to consider specific process characteristics and material properties to ensure the consistent and reliable production of internal threads. This aspect was explored by De Oliveira et al. [14], who delved into the impact of tooling and process parameters on the quality of internally formed threads. They observed that the optimization of thread quality can be achieved by selecting appropriate parameters such as forming speed, tap surface finish, and tap geometry. Moreover, in a separate investigation, Maciel et al. [15] ascertained the optimal revolutions for both forming and cutting external threads in a titanium alloy, relying on hardness testing and thread profile control.

In engineering, the setup of milling operations can be quite intricate due to the multitude of cutting parameters and tool geometrical aspects that demand consideration. Thread milling, when viewed from a geometric standpoint, represents a multifaceted 3D machining configuration, incorporating elements such as the toolpath, tool geometry, and the intricacies of the cutting process [16]. This particular technology carries a greater financial burden and necessitates access to suitable infrastructure and a high level of expertise for its effective application.

Over the past two decades, preliminary studies have focused on evaluating thread profile quality based on geometric errors [5]. In their work, Dogra and colleagues [17] delved into the evaluation of deviations between the tap’s feed rate and the thread pitch. Meanwhile, Freitas et al. [18] evaluated thread profile quality, employing the fundamental profile specifications of the ISO metric thread as outlined in ISO-68-1 and ISO 68-2, particularly during the threading of test samples.

A crucial factor in achieving a high-quality thread is a proper machined hole. The selection of hole manufacturing technology must consider not only its diameter but also factors encompassing material properties, depth, and the requisite levels of precision and functionality. Vigilant oversight of the resulting geometric and dimensional accuracy, alongside scrutiny of surface quality (including the identification of potential cracks or burrs) [19,20] proves indispensable, for it is impossible to produce a top-tier thread within an inadequately machined hole.

A specialized threaded joint involves the insertion of a screw into a thread directly within the thin-walled sheet of a manufactured structure. Nonetheless, during the manufacturing process, deviations in terms of thread shape and dimensions, or even damage, may manifest, ultimately resulting in suboptimal connections. Therefore, the appropriate selection of technology combinations for hole and thread production assumes vital importance. This forms the core focus of the experimental validations carried out within this study, as there is limited availability regarding information on this particular matter.

### Analysis of the Current Situation in Practice

This study examines the prevalent practices at ELMAX ŽILINA Slovakia., a company primarily employed in the production of electrical distribution boards and various sheet metal products, including those intended for use in the food industry. In the context of food sector products, it is common practice to employ corrosion-resistant steels, with a prevalent preference for austenitic chrome–nickel steel 1.4301, which is available in a range of thicknesses.

**Figure 1:**
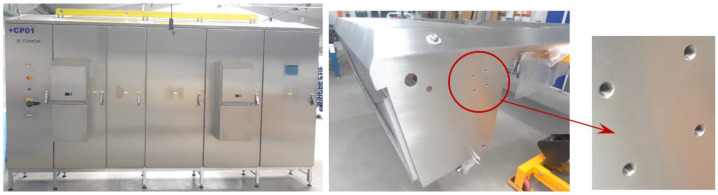
Electrical distribution cabinets monitored internal threads.

**Figure 2:**
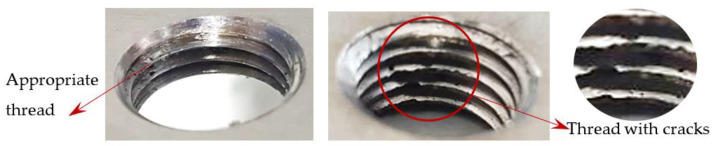
Internal thread M10 on the left without cracks in 5 mm thick sheet metal, and, on the right, with cracks in 10 mm thick sheet metal.

In the mentioned company, an analysis was carried out to solve a problem with making internal metric threads in semi-finished sheet metal with different thicknesses made of corrosion-resistant steel, as shown in Figure 1. While manufacturing the most frequently employed metric threads, evident deformations were observed after the production process, as depicted in Figure 2. These deformations emerge as cracks, rendering the threads partially non-functional and preventing them from meeting the standard-defined parameters.

The key issue lies in the frequent occurrence of potential thread damage, which poses a significant risk to both individual components and, in certain cases, the entire product itself. Frequently, these defective products are beyond repair, necessitating their removal from the manufacturing process. The need to remanufacture these products naturally results in higher production costs, longer production times, and a reduction in the actual profit margin of the product.

**Figure 3:**
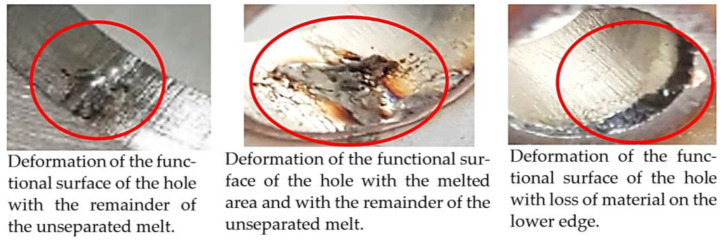
Holes made using LASER.

In the initial phase of our research, we discovered that holes created through laser machining displayed distinct defects (see Figure 3), leading to imprecise and irregular hole shapes. Furthermore, visible deformations within these holes were observed, posing a potential risk of imperfections in the internal threads manufactured within them.

The rationale behind employing laser machining for hole production in the aforementioned company, despite it encountering minor deformations, lies in its capacity to encompass all the outlines of the product, including the holes, in a single operation. This eliminates the need for additional drilling operations.

However, it is crucial to find the point where these deformations are still within acceptable limits and can be used to make high-quality, crack-free threads inside the holes that meet geometric standards.

When choosing a suitable advanced technology, it becomes especially crucial to analyze the problem of cracks and deformations in metric threads. These issues significantly diminish thread functionality, result in non-compliance with standards, and frequently render the product unusable, ultimately leading to its removal from the manufacturing process.

## 2. Materials and Methods

The mentioned company works with material 1.4301, which belongs to the category of materials characterized by specific properties acquired during the machining process. The chemical composition is shown in Table 1.

Steel 1.4301, known for its exceptional corrosion resistance, demonstrates resilience against water, steam, airborne moisture, and even edible acids. However, during the machining process, chromium–nickel steels themselves tend to undergo work hardening. This effect is predominantly undesirable and problematic, as it diminishes machinability and adversely impacts the final quality of the product [21].

The focus of this study is to examine the effects of modifications in manufacturing methods on the characteristics of threads manufactured from a particular material. The selection of utilized technologies is customized to meet the specific requirements of the company, ELMAX, inc. As a result, the choice of employed technologies is tailored to suit their requests. Threads were manufactured on sheets with varying thicknesses of 3, 5, 8, and 10 mm. Two distinct technologies were employed to create holes: progressive laser cutting technology and conventional drilling technology. Subsequently, the holes were utilized to create threads using two different methodologies: standard cutting technology and progressive forming technology.

The experimental measurements were set up following the specifications provided in Table 2. This encompassed the initial stages of preparing holes for threads and then inserting the threads within these holes. All necessary arrangements were made to facilitate subsequent comparisons and measurements.

**Figure 4:**
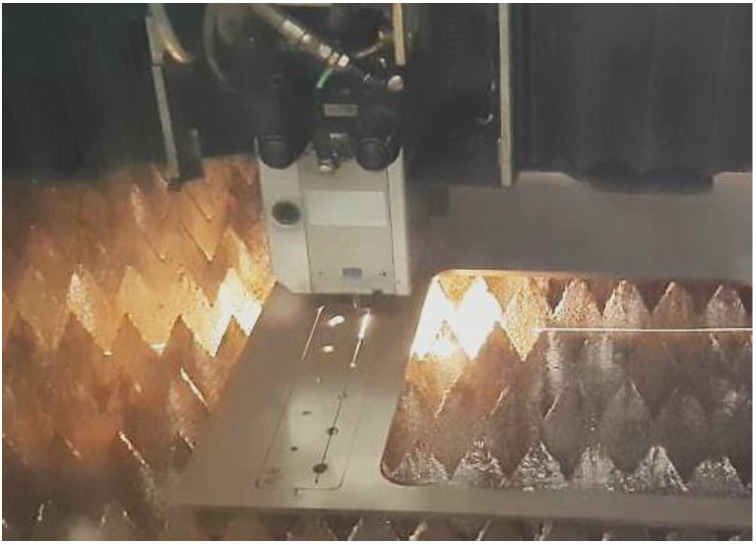
Laser production of one of the samples.

Samples, as shown in Figure 4, were prepared using a TruLaser 3030 machine, along with pre-cut openings of varying diameters, according to Table 3. In the course of the testing process, threads matching the dimensions commonly employed by the company were subsequently generated within these pre-prepared openings.

For tools, we employed DIN 338 drill bits made from high-speed steel, featuring a TiN coating and an angle of 118°. According to industry standards, these drill bits are suitable for drilling this specific type of steel. Cooling was facilitated through the use of Zubor 65 H Extra cooling emulsion, sourced from the German company ZEELER + GMELIN. The cutting parameters adhered closely to the recommendations of the manufacturer, maintaining a cutting speed (*v_c_*) of 14 m.min^−1^, while the feed rate varied in accordance with the diameter of the drill bit.

**Figure 5:**
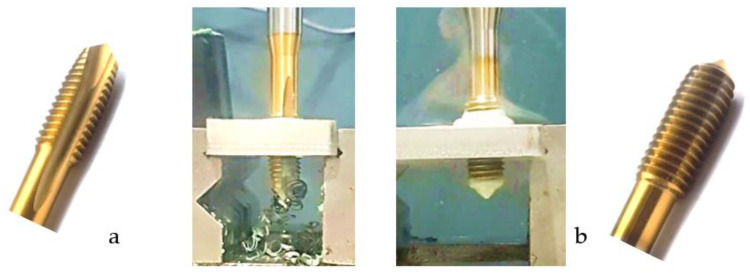
Manufacturing the M10 thread in an 8 mm thick sheet metal, (**a**)—by cutting and (**b**)—by forming.

Following the production of the holes, the production of metric threads through cutting was initiated. The MOSQUITO 300–600 threading machine was employed for this purpose. We utilized specially designed machine taps, namely DIN 371 HSS-E-PM-6H TiN taps. These taps are made from high-speed steel, enriched with a 5% cobalt alloy, and manufactured as sintered carbide with a coating, as illustrated in Figure 5a. For thread forming, DIN 2174 HSS-E-6HX TiN taps were chosen. These taps are composed of high-speed steel with a 5% cobalt addition and feature a coating, with one of them depicted in Figure 5b. To optimise the phase of the cutting efficiency, prevent potential tap binding, and reduce wear, we applied ARIANA cutting paste for threading.

Certain holes and, consequently, their corresponding threads, could not be produced due to technological limitations. Moreover, there were cases in which holes formed with deviations in their shape, resulting in negative consequences for the subsequent process of producing threads.

## 3. Results and Discussion

### 3.1. Visual Comparison of Functional Hole and Thread Surfaces

We conducted a visual inspection and quality evaluation for all the holes and threads. The functional surfaces of the holes are presented in Table 4, whereas those of the threads are detailed in Table 5, Table 6, Table 7 and Table 8.

Upon initial examination, several threads manufactured internally displayed evident visible signs of damage and lacked the appropriate thread profile. These thread profiles suffered from deformation, cracking, and insufficiency. Notably, these issues were most pronounced in threads produced using thread-forming technology within holes produced using laser cutting, regardless of sample thickness. This predicament arises from the thermally affected zone on the functional surfaces of the holes, a byproduct of the laser-cutting hole production process. Consequently, it can be deduced that employing the forming process within holes created using laser cutting is an unfavourable choice, given the considerably larger-than-expected thermally affected zone. Furthermore, imperfections in the thread profile were only apparent in threads produced by cutting within holes produced using laser cutting, specifically in samples with thicknesses of 8 mm and 10 mm and for M8 and M10 threads. This inconsistency can be attributed to the suboptimal geometry of holes created by laser cutting in thicker samples and those with ø 8.5 and ø 7 diameters.

### 3.2. Measurement of Residual Stresses

**Figure 6:**
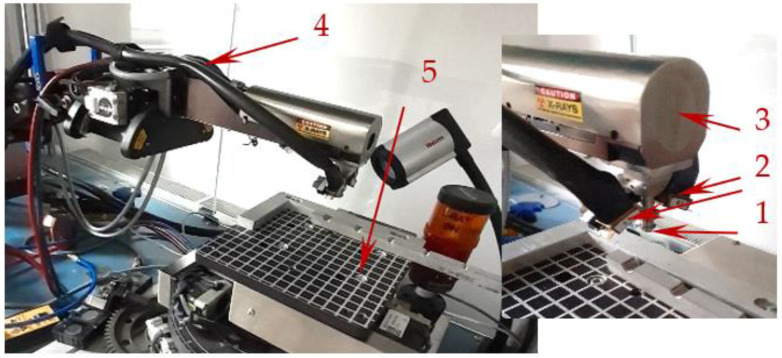
Device for measuring residual stresses Proto iXRD (laboratory setup): 1—collimator directing the X-ray beam; 2—a pair of detectors capturing the diffraction cone; 3—X-ray tube with Cr target; 4—Cobralink ^®^ flexible measuring arm; 5—adjustable and rotating table.

The process of measuring residual stresses through X-ray diffraction can be explained with the Proto iXRD device (Figure 6). This equipment enables the measurement of residual stresses in all crystalline materials and, due to its design, is suitable for use both in laboratory settings and in real manufacturing processes [22,23]. Residual stress evaluation for the produced holes and threads was carried out utilizing X-ray diffraction on the Proto iXRD measurement device.

The same measurement parameters were used when measuring residual stresses in individual holes and threads: a fixed number of measurements (angles) at one position, ±30° with 15 angular positions, X-ray tube: Mn_K(α), Collimator: 1mm diameter, filter: Cr, voltage: 20 KV, current: 4mA, and beta oscillation: 3°.

**Figure 7:**
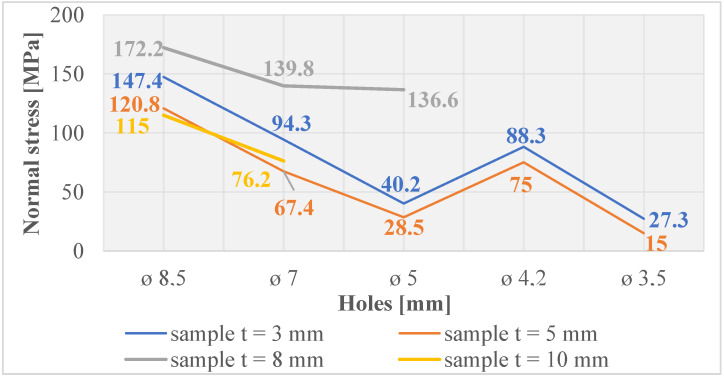
Normal stress in subsurface layers of holes produced using laser cutting.

**Figure 8:**
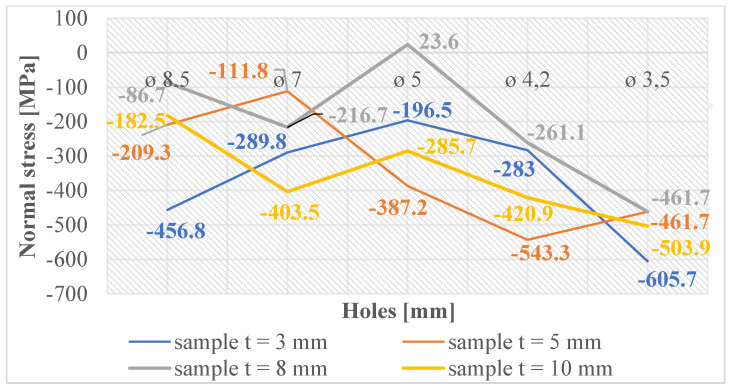
Normal stress in subsurface layers of holes produced by drilling.

**Figure 9:**
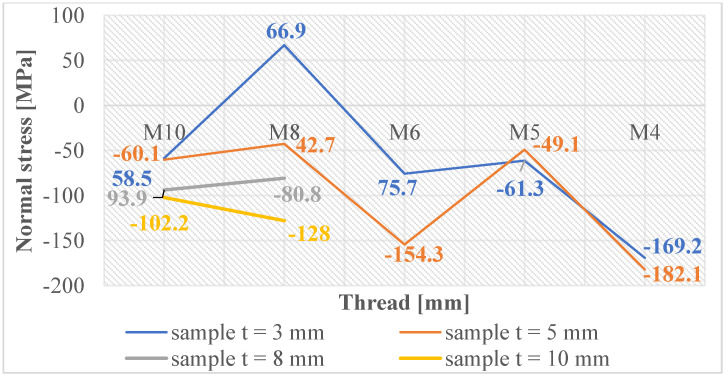
Normal stress in subsurface layers of threads produced by cutting in the holes produced using laser cutting.

**Figure 10:**
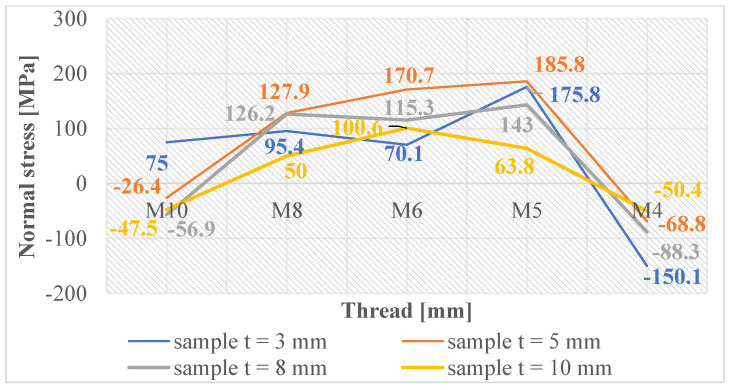
Normal stress in subsurface layers of threads produced by cutting in the holes produced by drilling.

**Figure 11:**
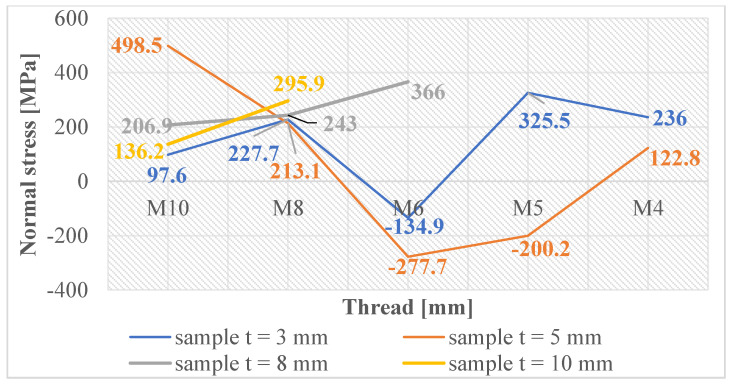
Normal stress in subsurface layers of threads produced by forming in the holes produced using laser cutting.

**Figure 12:**
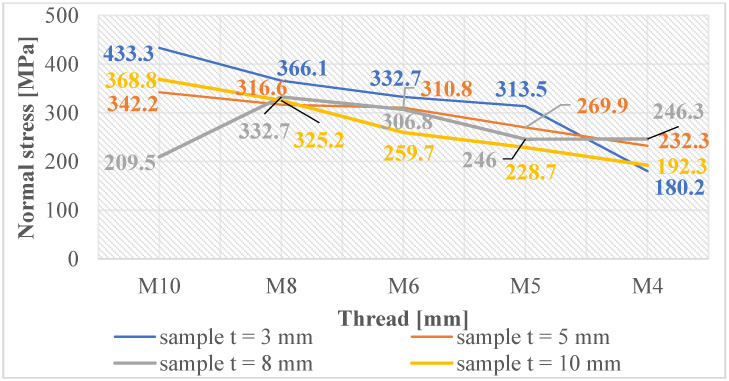
Normal stress in subsurface layers of threads produced by forming in the holes produced by drilling.

Normal and shear stresses were measured in all holes and threads. The recorded values were used to calculate nominal (average) values, and graphical representations were generated for better visualization, as depicted in Figure 7, Figure 8, Figure 9, Figure 10, Figure 11 and Figure 12.

The measured values in the holes created using laser cutting (Figure 7) show low values above the zero axis, confirming minimal normal tensile stresses close to the material’s equilibrium state. Figure 8 reveals that holes created by drilling are below the zero axis, indicating significant normal compressive stresses, implying material strengthening in subsurface layers.

Figure 9 provides an opportunity for observation, revealing that the production of threads through the process of cutting into holes generated using laser cutting typically results in minor normal compressive stresses. However, it is worth noting that an exception is observed in the case of the M8 thread. On the other hand, it can be observed that threads formed within drilled holes (as depicted in Figure 10) experience normal compressive stresses in the case of M4 and M10 threads. While the M8, M6, and M5 threads demonstrate normal tensile stresses.

One could argue that the production of threads by cutting into pre-drilled holes induces larger normal compressive stresses in these holes, thereby creating a strengthened layer. However, when threads are cut through this layer, it loses its integrity. This means that most threads have opposing normal tensile stresses that are low and close to the equilibrium state of the material.

For threads made by cutting into holes made using laser cutting, where there were initially small normal tensile stresses, the process of cutting the threads changed these values so that most of the stresses in the threads were normal compressive stresses. Nonetheless, these stress levels are closely aligned with the equilibrium state of the material, thereby avoiding substantial modifications in the subsurface layers and maintaining the material free from extreme internal stresses.

In Figure 11, the graph reveals that the majority of measured values indicate the presence of normal compressive stresses in the subsurface layers of threads produced by forming holes created by laser cutting. These stress values exhibit some variability, which can be attributed to the thermally affected zone generated during the laser-cutting process. Conversely, when examining the measured values of threads produced by forming holes created by drilling, as depicted in Figure 12, we observe that these values are closely grouped. This clustering unequivocally illustrates that only normal compressive stresses were induced in the subsurface layers of these threads, with a less pronounced thermal influence during hole production.

### 3.3. Measurement of Thread Parameters

The thread profiles of all manufactured threads were assessed using the CONTOURECORD 1700SD3 measuring device, employing a probing tip with a 25 µm radius for the measurements. The instrument recorded the profiles of all threads, allowing for the evaluation of two fundamental thread parameters. The values of these parameters, following the STN ISO 262:2000 (01 4010) standard, are detailed in Table 9.

Due to the large volume of data generated for evaluating measurable thread parameters, we have chosen to present only selected measurements and summarize the results in a table for better comprehension.

### 3.4. Results of Measurements of Thread Parameters Produced by Cutting

**Figure 13:**
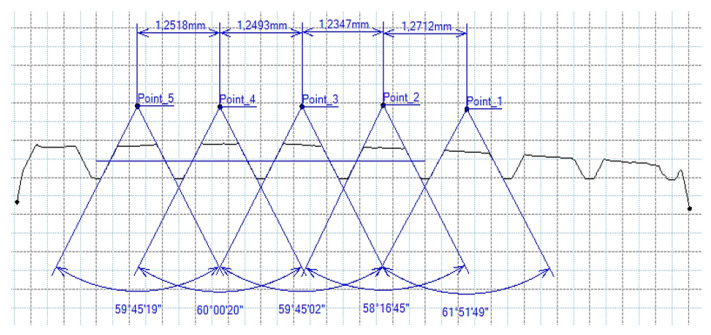
The M8 thread is produced by cutting into a hole made by a laser in sheet metal with a thickness of 10 mm.

**Figure 14:**
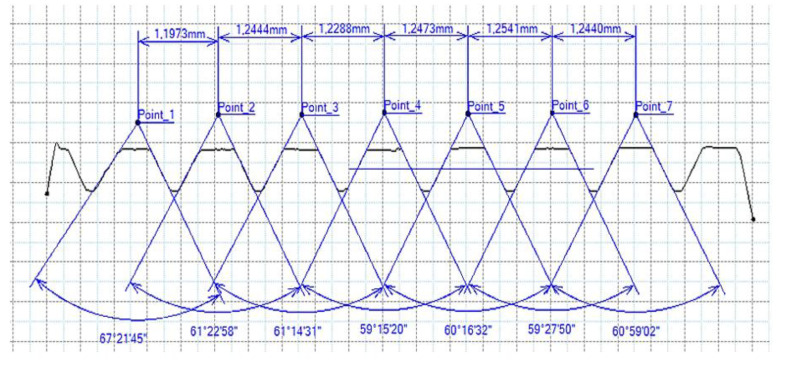
The M8 thread is produced by cutting into a hole made by drilling in sheet metal with a thickness of 10 mm.

Figure 13 and Figure 14 provide a basis for comparing two sets of measurements for internally threaded holes with identical dimensions. These holes were created by cutting into the same sample thickness but using different hole production technologies. This comparison leads to the conclusion that the threads produced by cutting into holes created by laser cutting (Figure 13) are deemed unsatisfactory. This is evident because the two thread profiles do not conform to the required specifications, and it was not feasible to measure the parameters on these profiles. Additionally, a visual inspection reveals that the thread heights, and, consequently, the major diameter of the thread, are smaller when compared to threads produced by cutting into holes created by drilling (Figure 14). Hence, despite the measured parameter values falling within acceptable limits, the threads produced by cutting into holes created using laser cutting are considered unsatisfactory.

### 3.5. Results of Measurements of the Parameters of Threads Produced by Forming

**Figure 15:**
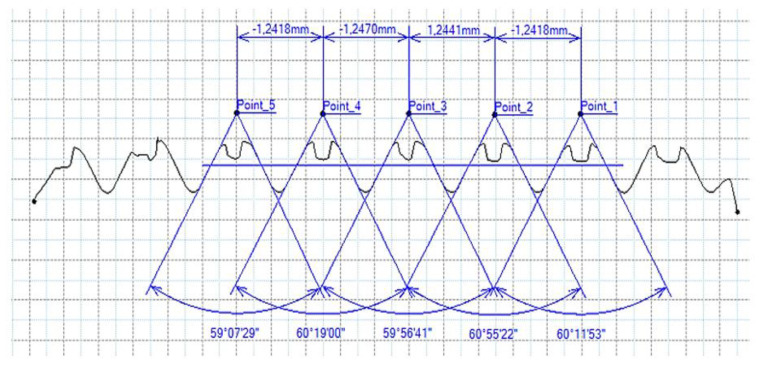
The M8 thread is produced by forming into hole made by a laser, a sheet metal thickness of 10 mm.

**Figure 16:**
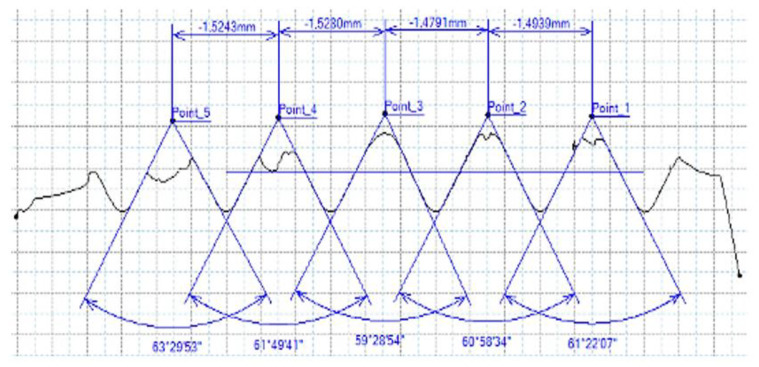
The M8 thread is produced by forming into hole made by drilling, a sheet metal thickness of 10 mm.

In Figure 15 and Figure 16, we can compare two sets of contour measurements for internally threaded holes with identical dimensions. These threads were created within the same 10 mm thick sample but using different hole production technologies. This comparison clearly shows that the shape and parameters of the threads produced by forming within holes created using laser cutting (Figure 15) are unsatisfactory, unlike the threads produced by forming within holes created by drilling (Figure 16).

Measurements revealed that all internally threaded holes made by forming within holes made by laser cutting were unsatisfactory, except for one case. Upon evaluation, we identified deficiencies such as imperfect shapes with deformations or unsatisfactory parameters. Hence, it is evident that manufacturing using these technologies for both the hole and thread lacks precision, and it is not feasible to produce high-quality threads in this manner. Consequently, this approach cannot be employed in practical applications at all.

Using data from the contour measuring device, we assessed all threads based solely on their profile contours, specifically examining their shape, potential cracks, deformations, and the completeness of the thread profile. Threads with noticeable shape imperfections were categorized as unsatisfactory, while those with acceptable shapes were considered satisfactory, as indicated in Table 10.

## 4. Conclusions and Benefits

All the threads that were produced and measured, and subsequently assessed for thread quality, residual stress levels within the threads, thread profile shape, and measured parameters (thread profile angle α and thread pitch P), were processed following the guidelines outlined in Table 11.

Subsequently, individual threads were evaluated as either good ✔, very good ✔✔, or poor ✖ (see Table 12).

The finalized results of the conducted experimental verifications and their evaluations are presented in Table 12. This table clearly illustrates that the most satisfactory internal threads can be achieved by employing thread-cutting technology within holes created by drilling. However, it is unviable to produce larger threads like M8 and M10 in thinner sheets with thicknesses of t = 3 mm and t = 5 mm using this approach. Furthermore, manufacturing a small M4 thread with a thickness of 10 mm is also unfeasible.

The feasibility of producing threads within holes created using laser cutting, including the suitable thread sizes, material thicknesses, and production methods, has also been investigated. Based on the findings, it is feasible to create high-quality threads by cutting into holes created by laser cutting, particularly for the M4, M5, and M6 threads with thicknesses of t = 3 mm and t = 5 mm. Nevertheless, considering the continually unsatisfactory outcomes of all previous threads generated using this technique, it is implausible to consistently deem the threads formed within holes created using laser cutting as acceptable.

The threads produced by forming within holes created by drilling were satisfactory only for thicknesses of t = 8 mm and t = 10 mm.

The primary contribution of this study lies in the obtained results, which serve as valuable recommendations for selecting suitable manufacturing technologies for the production of the analyzed metric internal threads (M4, M5, M6, M8, M10) within the material 1.4301, considering various thicknesses ranging from 3 mm to 10 mm, specifically tailored to the needs of the aforementioned company.

These recommendations and insights will be put into practice at ELMAX ŽILINA inc. and subjected to further validation. However, it is important to note that the results have broader applicability and can be useful in a wide range of practical scenarios where the manufacturing technologies tested in the conducted verifications are employed.

## Figures and Tables

**Table 1:** Chemical composition of material 1.4301 according to EN 10088.

Chemical Composition [%]						
C	Cr	Mn	Ni	P	S	Si
0.07	17–20	2	9–11.5	0.045	0.03	1
**Mechanical Properties**						
Yield strength R_e_ [MPa]		Tensile strength R_m_ [MPa]		Hardness [HB]		Ductility A [%]
min 230		540–750		160–210		45

**Table 2:** Sample production process table.

	Product	Production Process	Machine	Tool
Exp. 1	Hole	Laser	TRULASER 3030	Laser Beam
	Thread	Cutting	Thread Cutter MOSQUITO 300–600	HSS-E-PM 6H TiN DIN 371
Exp. 2	Hole	Drilling	HURCO VMX30t	HSS TiN DIN 338
	Thread	Cutting	Thread Cutter MOSQUITO 300–600	HSS-E-PM 6H TiN DIN 371
Exp. 3	Hole	Laser	TRULASER 3030	Laser Beam
	Thread	Forming	Thread Cutter MOSQUITO 300–600	HSS-E 6HX TiN DIN 2174
Exp. 4	Hole	Drilling	HURCO VMX30t	HSS TiN DIN 338
	Thread	Forming	Thread Cutter MOSQUITO 300–600	HSS-E 6HX TiN DIN 2174

**Table 3:** Laser production of one of the samples.

Thread Size	Hole Diameter for Thread Made by Cutting [mm]	Hole Diameter for Thread Made by Forming [mm]
M4	ø 3.5	ø 3.7
M5	ø 4.2	ø 4.6
M6	ø 5.0	ø 5.5
M8	ø 7.0	ø 7.4
M10	ø 8.5	ø 9.3

**Table 4:** Functional hole surfaces in the cut for various sheet metal thicknesses and hole diameters.

				Hole for Thread				
				M4	M5	M6	M8	M10
Sheet thickness	3 mm	Technology	Laser	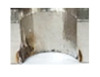	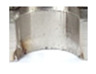	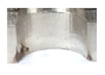	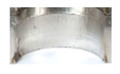	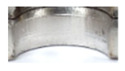
			Drilling	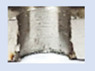	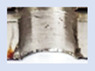	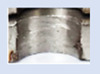	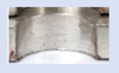	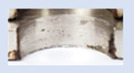
	5 mm		Laser	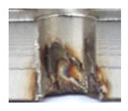	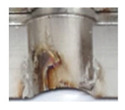	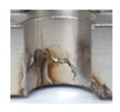	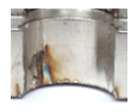	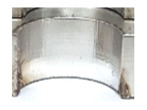
			Drilling	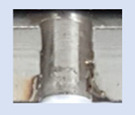	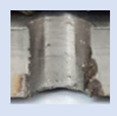	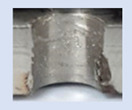	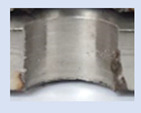	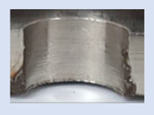
	8 mm		Laser			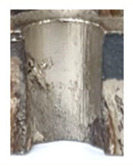	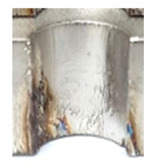	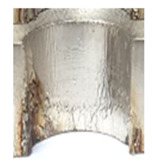
			Drilling	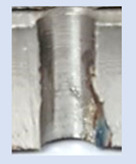	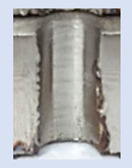	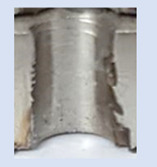	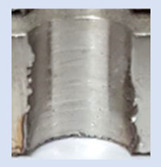	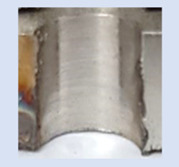
	10 mm		Laser				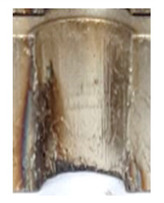	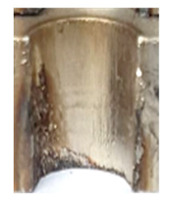
			Drilling	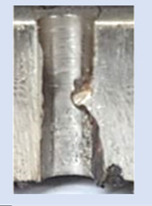	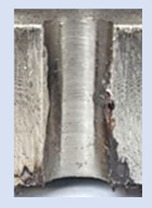	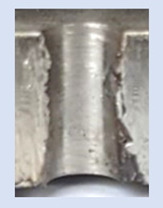	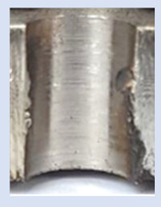	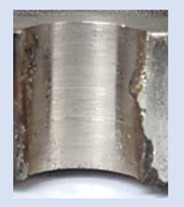

**Table 5:** Functional thread surfaces in the cut for a sheet metal thickness of 3 mm.

					Thread				
					M4	M5	M6	M8	M10
Sheet thickness 3 mm	Technology of hole production	Laser	The technology of thread production	Cut	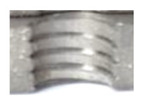	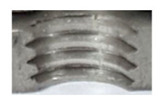	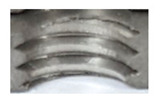	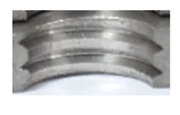	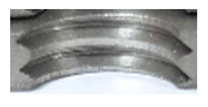
				Formed	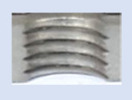	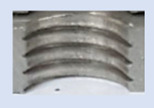	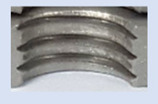	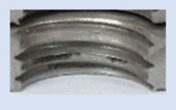	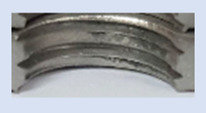
		Drilling		Cut	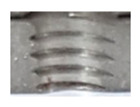	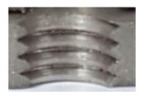	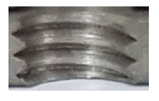	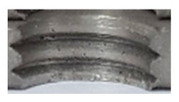	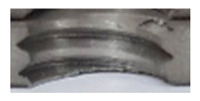
				Formed	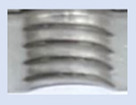	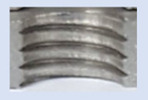	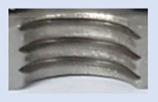	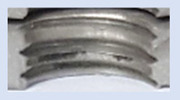	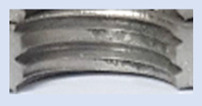

**Table 6:** Functional thread surfaces in the cut for a sheet metal thickness of 5 mm.

					Thread				
					M4	M5	M6	M8	M10
Sheet thickness 5 mm	Technology of hole production	Laser	The technology of thread production	Cut	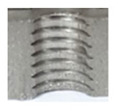	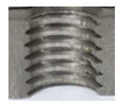	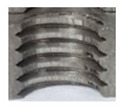	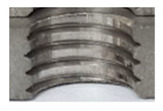	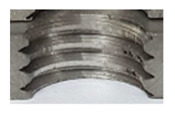
				Formed	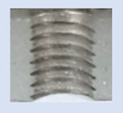	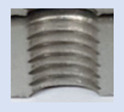	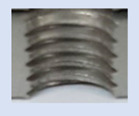	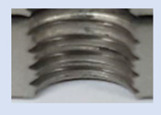	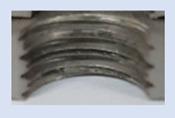
		Drilling		Cut	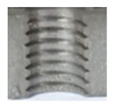	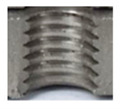	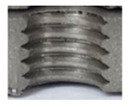	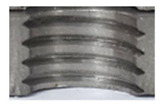	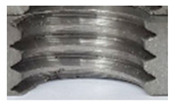
				Formed	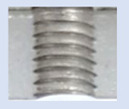	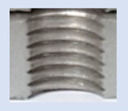	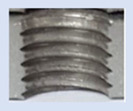	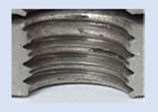	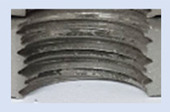

**Table 7:** Functional thread surfaces in the cut for a sheet metal thickness of 8 mm.

					Thread				
					M4	M5	M6	M8	M10
Sheet thickness 8 mm	Technology of hole production	Laser	The technology of thread production	Cut				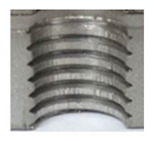	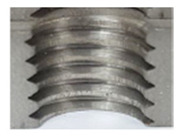
				Formed			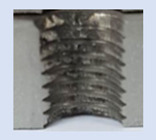	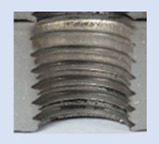	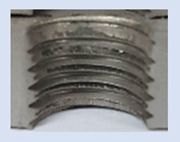
		Drilling		Cut	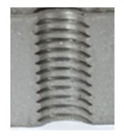	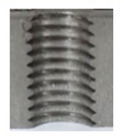	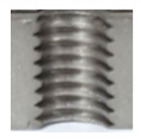	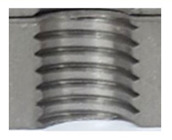	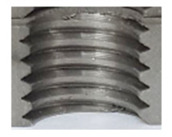
				Formed	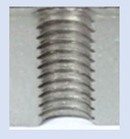	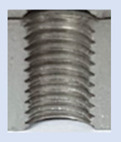	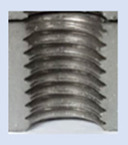	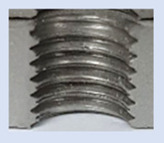	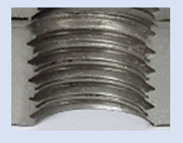

**Table 8:** Functional thread surfaces in the cut for a sheet metal thickness of 10 mm.

					Thread				
					M4	M5	M6	M8	M10
Sheet thickness 10 mm	Technology of hole production	Laser	The technology of thread production	Cut				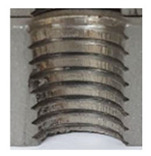	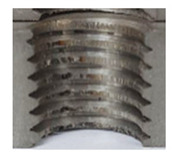
				Formed				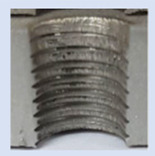	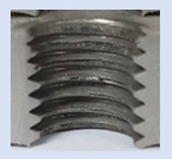
		Drilling		Cut	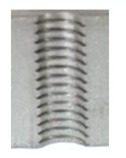	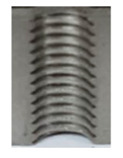	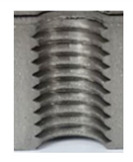	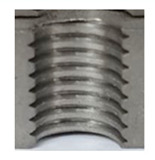	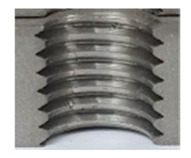
				Formed	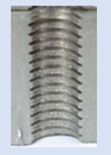	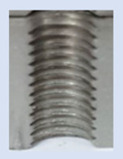	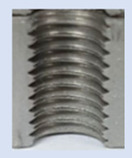	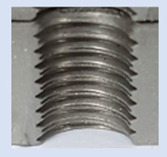	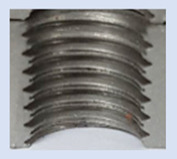

**Table 9:** Table of nominal values of two measured parameters on manufactured threads.

**Thread size [mm]**	M4	M5	M6	M8	M10
**Spacing P [mm]**	0.7	0.8	1	1.25	1.5
**Thread profile angle α [°]**	60				

**Table 10:** Evaluation of the measured outputs of the thread profile.

Sheet Thickness	Production Technology		Thread				
	Hole	Thread	M4	M5	M6	M8	M10
3 mm	Laser	Cut	✔	✔	✔	✔	✔
		Formed	✖	✖	✖	✖	✖
	Drilling	Cut	✔	✔	✔	✔	✔
		Formed	✖	✖	✖	✖	✖
5 mm	Laser	Cut	✔	✔	✔	✔	✔
		Formed	✔	✖	✖	✖	✖
	Drilling	Cut	✔	✔	✔	✔	✔
		Formed	✖	✖	✖	✖	✖
8 mm	Laser	Cut	*-*	-	-	✖	✖
		Formed	*-*	-	✖	✖	✖
	Drilling	Cut	✔	✔	✔	✔	✔
		Formed	✔	✔	✔	✔	✔
10 mm	Laser	Cut	*-*	-	*-*	✖	✖
		Formed	*-*	-	*-*	✖	✖
	Drilling	Cut	✔	✔	✔	✔	✔
		Formed	✔	✔	✔	✔	✔

**Table 11:** Comprehensive evaluation of form-fitting threads.

Thread Marking	Thread M10 into Drilled Hole, t = 10 mm		Thread M8 into Drilled Hole, t = 10 mm	
	**Rezaný**	**Tvárnený**	**Rezaný**	**Tvárnený**
Normal stresses in the hole [MPa]	−182.5 ± 19.4	-	−403.5 ± 24.1	-
Shear stress in the hole [MPa]	−32.9 ÷ 10	-	−1.3 ± 12.4	-
Normal stresses in the thread [MPa]	−47.5 ± 35.8	368.5 ± 106.5	50 ± 30.7	332.7 ± 37.1
Shear stresses in the thread [MPa]	−11 ± 17.2	−40.8 ± 51.2	−17.6 ± 14.7	−11 ± 17.8
Arithmetic diameter of thread profile angles α [°]	61° 16	59° 30′	61° 26′	60° 7′
Arithmetic diameter of thread pitches P [mm]	1.48	1.49	1.24	1.24
Evaluation	good	very good	good	very good

**Table 12:** Evaluation of the quality of individual produced threads.

	M4	M5	M6	M8	M10
Internal threads produced by cutting into holes made by drilling					
t = 3 mm	✔	✔	✔	✖	✖
t = 5 mm	✔✔	✔✔	✔	✖	✖
t = 8 mm	✔	✔	✔✔	✔	✔
t = 10 mm	✔	✔✔	✔✔	✔	✔
Internal threads produced by cutting into holes made by laser cutting					
t = 3 mm	✔✔	✔✔	✔✔	✖	✖
t = 5 mm	✔✔	✔✔	✔	✖	✖
t = 8 mm	✖	✖	✖	✖	✖
t = 10 mm	✖	✖	✖	✖	✖
Internal threads produced by forming into holes made by drilling					
t = 3 mm	✖	✖	✖	✖	✖
t = 5 mm	✖	✖	✖	✖	✖
t = 8 mm	✖	✔✔	✔✔	✔	✔✔
t = 10 mm	✔✔	✔✔	✔✔	✔✔	✔✔
Internal threads produced by forming into holes made by laser cutting					
t = 3 mm	✖	✖	✖	✖	✖
t = 5 mm	✔	✖	✖	✖	✖
t = 8 mm	✖	✖	✖	✖	✖
t = 10 mm	✖	✖	✖	✖	✖

## Data Availability

The data are available in a publicly accessible repository.
